# Serum APE1 as a predictive marker for platinum-based chemotherapy of non-small cell lung cancer patients

**DOI:** 10.18632/oncotarget.13030

**Published:** 2016-11-02

**Authors:** Shiheng Zhang, Le He, Nan Dai, Wei Guan, Jinlu Shan, Xueqin Yang, Zhaoyang Zhong, Yi Qing, Feng Jin, Chuan Chen, Yuxin Yang, Hongyi Wang, Laura Baugh, Gianluca Tell, David M. Wilson, Mengxia Li, Dong Wang

**Affiliations:** ^1^ Cancer Center of Research Institute of Surgery, Daping Hospital, Third Military Medical University, Chongqing 400042, China; ^2^ Laboratory of Molecular Biology and DNA repair, Department of Medical and Biological Sciences, University of Udine, Udine 33100, Italy; ^3^ Laboratory of Molecular Gerontology, National Institute on Aging, Intramural Research Program, National Institutes of Health, Baltimore, MD 21224, USA; ^4^ Department of Pathology, Baylor University Medical Center, Dallas, TX 75246, USA; ^5^ Tianjin HUABOTE Biotechnological Inc., Tianjin 300350, China

**Keywords:** APE1, NSCLC, chemotherapy, biomarker

## Abstract

**Purpose:**

To define the role of the DNA repair protein apurinic/apyrimidinic endonuclease 1 (APE1) in predicting the prognosis and chemotherapeutic response of non-small cell lung cancer patients receiving platinum-containing chemotherapy.

**Results:**

Our investigations found that serum APE1 level was significantly elevated in 229 of 412 NSCLC patients and correlated with its level in tissue (*r*^2^ = 0.639, *p* < 0.001). The elevated APE1 level in both tissue and serum of patients prior to chemotherapy was associated with worse progression-free survival (HR: 2.165, *p* < 0.001, HR: 1.421, *p* = 0.012), but not with overall survival. After 6 cycles of chemotherapy, a low APE1 serum level was associated with better overall survival (HR: 0.497, *p* = 0.010).

**Experimental Design:**

We measured APE1 protein levels in biopsy tissue from 172 NSCLC patients and sera of 412 NSCLC patients receiving platinum-based chemotherapy by immunohistochemistry and a newly established sensitive and specific enzyme-linked immunosorbent assay, respectively. APE1 levels in sera of 523 healthy donors were also determined as control.

**Conclusions:**

Our studies indicate that APE1 is a biomarker for predicting prognosis and therapeutic efficacy in NSCLC. The chemotherapy-naïve serum APE1 level, which correlated with its tissue level inversely associated with progression-free survival of platinum-containing doublet chemotherapy, whereas post-treatment serum APE1 level was inversely associated with overall survival.

## INTRODUCTION

Current pan-cancer analyses have revealed that most cancers are driven by mutations in a subset of genes, highlighting the role of mutagenesis and genomic instability in the process of carcinogenesis [1]. In addition, during cancer treatment, the induction of DNA lesions is the predominant cytotoxic mechanism of both chemotherapy and radiotherapy. Thus, as guardians of the genome, DNA repair mechanisms serve to protect against the biological consequences of both endogenous and externally-induced DNA damage. Alterations in DNA repair during the evolution of cancer cells can promote carcinogenesis, as well as regulate resistance to genotoxic chemo- and radio-therapies [2]. Thus, further information about the dynamics of DNA repair in cancer etiology presents an opportunity, not only to better understand the development of cancer, but also to advance the application of molecular-driven therapeutics.

Numerous studies have revealed a potential role for DNA repair genes/proteins as biomarkers in different types of cancer, including non-small cell lung cancer (NSCLC) (reviewed in [3]). Notably, NSCLC is a cancer for which genotyping of tumor cells, particularly with respect to epidermal growth factor receptor (EGFR) mutations, has demonstrated a remarkable level of success in treatment planning and efficacy (reviewed in [4]). An ideal predictive biomarker assay for tumor screening or for monitoring therapeutic efficacy must be reproducible, sensitive, specific, cost-effective and able to be executed rapidly. Since DNA repair proteins predominantly reside in the nucleus, most proteins have only been detected in biopsy tissues and therefore do not meet the criteria for a desirable biomarker. To combat this problem, efforts have been made to detect autoantibodies in plasma and serum against DNA repair-associated proteins: including the tumor suppressor p53 [5, 6], replication protein A 32 [7], the DNA strand break response protein poly(ADP-ribose) polymerase 1 (PARP1), the breast cancer associated proteins 1 and 2 (BRCA1/2) [8], the human mismatch repair proteins, MutS homolog 2 (hMSH2) and postmeiotic segregation increased 1 (hPMS1) [9], and the DNA non-homologous end-joining factor Ku [10]. However, the production of autoantibodies against DNA repair-associated proteins depends on antigen exposure and the immune status of the subjects. Although p53 protein can be detected in serum [11], to our knowledge, until recently, no core DNA repair enzyme, other than apurinic/apyrimidinic endonuclease 1 (APE1), has been reported to be found in serum.

APE1 is an essential, multi- functional protein possessing both DNA repair and transcriptional regulatory activities. Most notably, it participates in the base excision repair (BER) pathway, and its activity accounts for over 95% of the total AP endonuclease activity in human cells, making it critical to overall BER capacity (reviewed in [12]). In addition, APE1 regulates the DNA binding affinity of several important transcription factors, including NF-κB, AP-1 and p53 through redox-dependent and -independent mechanisms. APE1 protein expression has been measured primarily in formalin-fixed, paraffin-embedded (FFPE) biopsy tissue sections from patients and matched controls or pre-cancerous tissue using immunohistochemistry (IHC). Collectively, studies from our group and others have shown that elevated nuclear APE1 protein levels, as well as cytoplasmically accumulated APE1, are associated with poor prognosis and chemoresistance (reviewed in [12]).

In addition to direct tissue detection of APE1, this protein has also, surprisingly, been indirectly detected through the presence of anti-APE1 autoantibodies discovered initially in the context of systemic lupus erythematosus (SLE) [13]. Based on this observation, we speculated that APE1 protein could also be present in human serum under other pathological conditions. Our laboratory was the first to report the presence of APE1 in serum from peripheral blood samples of NSCLC patients [14]. Although statistical significance was not reached in that study due to the relatively small sample size, we still observed an elevation of serum APE1 (from hereon referred to as sAPE1) levels in NSCLC patients associated with advanced stages of the disease.

Based on the above preliminary observations, we investigated herein APE1 as a biomarker for NSCLC diagnosis and response to platinum-containing chemotherapy. We initially measured APE1 protein levels in biopsy tissue of NSCLC patients receiving platinum-based doublet chemotherapy by IHC, with the goal of correlating APE1 tissue expression with therapeutic response. To improve our analyses, we increased our sample size for greater power, and in order to accurately measure trace amounts of sAPE1, we established a sophisticated enzyme-linked immunosorbent assay (ELISA) with high sensitivity and a wide linear-range of detection. Our investigations indicate that (i) increased APE1 protein levels in NSCLC biopsy tissue are correlated with a decreased sensitivity to chemotherapy and (ii) sAPE1 is significantly elevated in NSCLC patients and enhances diagnostic power of carcinoembryonic antigen (CEA). In addition, elevated APE1 levels are associated with shorter progression-free survival (PFS) after chemotherapy, suggesting that APE1 is a promising biomarker for monitoring therapeutic efficacy.

## RESULTS

### Expression of APE1 protein in NSCLC tissue

**Figure 1:**
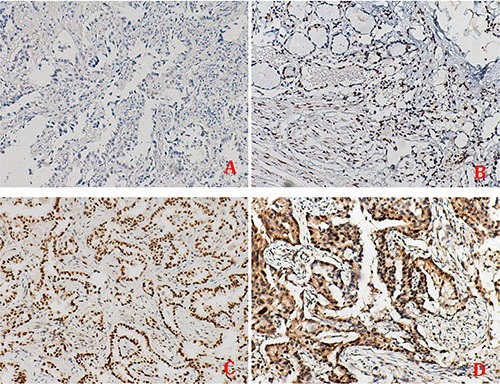
The tissue expression of APE1 in non-small cell lung cancer APE1 protein was scored for four categories: (**A**) score 0, (**B**) score 1+, (**C**) score 2+ and (**D**) score 3+, and was detected in three subcellular patterns in tumor tissues: the nuclear, the cytoplasm and both in nucleus and cytoplasm. Original magnification: ×100.

APE1 expression analysis was conducted on a cohort of 172 advanced NSCLC patients, for which the clinicopathological characteristics are summarized in Table 1. IHC revealed that APE1 nuclear expression is present in 73.25% of the tissue samples, with exclusive nuclear expression in 33.72% and dual expression in both the nucleus and cytoplasm in 39.53% of the tissues (representative images shown in Figure 1).

**Table 1:** The demographic and clinical characteristics of tissue cohort

Characteristics	Number
Age	
Median (Range)	60.46 (34–84)
< 60	82 (47.67%)
> = 60	90 (52.33%)
Gender	
Female	55 (31.98%)
Male	117 (68.02%)
Stage	
III	63 (36.63%)
IV	109 (63.37%)
Histopathology	
Adenocarcinoma	106 (61.63%)
Squamous cell carcinoma	34 (19.77%)
Mixed/other NSCLC	32 (18.60%)
Smoking status	
Never	69 (40.12%)
Ever-smoker	103 (59.88%)
Performance status	
0	103 (59.88%)
1	56 (32.56%)
2	13 (7.56%)
APE1 expression score	
0	19 (11.04%)
1	27 (15.70%)
2	65 (37.79%)
3	61 (35.47%)

### Tissue APE1 expression level is inversely associated with platinum-based chemotherapy response

**Figure 2:**
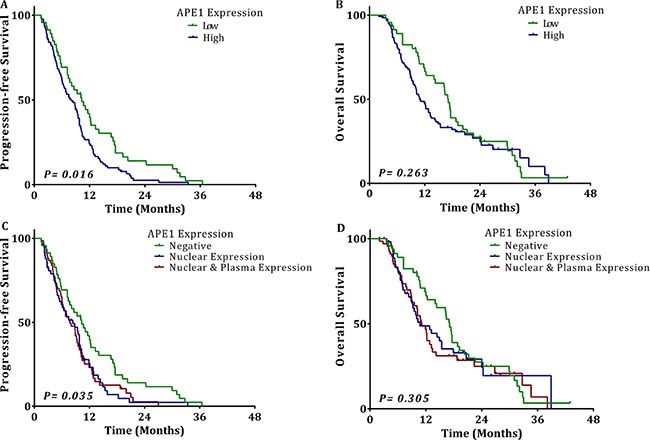
The association of tissue APE1 expression with outcomes of NSCLC Kaplan-Meier univariate analysis of (**A**) progression-free survival (10.3 vs. 8.0 months, *p* = 0.016) and (**B**) overall survival (17.1 vs. 10.9 months, *p* = 0.263) for patients with NSCLC showing low expression of tissue APE1 and patients with NSCLC showing high expression of tissue APE1, (**C**) progression-free survival (8.4 vs. 7.7 months, *p* = 0.035) (**D**) overall survival (10.7 vs. 11.1 months, *p* = 0.305) for patients with NSCLC showing negative tissue expression and patients with NSCLC showing exclusive nuclear expression or combined nuclear/cytoplasmic expression of tissue APE1.

Among the 172 patients, 136 (79.07%) received taxol/docetaxel plus cisplatin (TP) regimens and 36 (20.93%) received gemcitabine plus cisplatin (GP) regimens with an average cycle number of four (range from 2 to 8). The responses to the chemotherapy included: 2 complete response (CR) (1.16% of evaluated patients), 53 partial response (PR) (30.81%), 65 stable disease (SD) (37.79%), and 52 progressive disease (PD) (30.23%). The objective response rate (ORR) in the group with high APE1 expression was 25.40%, while the ORR was 50.00% in the group with low APE1 expression (OR 5.233; 95% CI 2.029-13.443; *p* = 0.001, shown in Table 2). Kaplan–Meier survival curves showed that the APE1 negative group had a significantly longer median PFS (10.3 vs. 8.0 months, *p* = 0.016). Although not statistically significant, overall survival (OS) is longer in the APE1 negative group as well (17.1 vs. 10.9 months, *p* = 0.263) (Figure 2). However, NSCLC patients with either exclusively nuclear or combined nuclear/cytoplasmic expression of APE1 had almost the same median PFS (8.4 vs. 7.7 months) and OS (10.7 vs. 11.1 months). In addition, the APE1 low-expression group treated with TP regimens vs. GP regimens had almost the same response rate (48.48% vs. 53.85%, *p* = 0.175), median PFS (11.1 vs. 7.4 months, *p* = 0.277) and OS (17.0 vs. 17.5 months, *p* = 0.742). These data suggest that APE1 is a predictive factor for platinum sensitivity, but not for the other agents (taxol/docetaxel and gemcitabine) used in the different chemotherapy regimens. A multivariate Cox regression analysis showed that high tissue APE1 expression is independently associated with shorter PFS (HR 2.165; 95% CI: 1.455–3.221; *p* < 0.001) and OS (HR 2.543; 95% CI 1.639–3.947; *p* < 0.001) (Table 2).

**Table 2:** Association of APE1 tissue subcellular location with the outcomes of NSCLC receiving chemotherapy

				Treatment response			Progression-free survival				Overall survival	
	Number of cases	CR+PR	SD+PD	ORR (%)	Multivariable Logistic analysis		Months	Multivariable COX regression		Months	Multivariable COX regression	
					OR (95% CI)	*P* value		HR (95% CI)	*P* value		HR (95% CI)	*P* value
APE1 tissue expression												
Low	46	23	23	50.00			10.30			17.10		
High	126	32	94	25.40	5.233 (2.029–13.443)	0.001	8.00	2.165 (1.455–3.221)	< 0.001	10.90	2.543 (1.639–3.947)	< 0.001
APE1 tissue subcellular location												
Low expression	46	23	23	50.00			10.30			17.10		
Nuclear only	58	15	43	25.90	4.338 (1.522–12.366)	0.006	8.40	2.210 (1.403–3.481)	0.001	10.70	2.481 (1.484–4.147)	0.001
Nuclear & cytoplasm	68	17	51	25.00	4.798 (1.758–13.093)	0.002	7.70	2.130 (1.380–3.288)	0.001	11.10	2.589 (1.605–4.177)	< 0.001
Treatments in APE1 low expression												
Paclitaxel	33	16	17	48.48			11.10			17.00		
Other	13	7	6	53.85	0.353 (0.078–1.590)	0.175	7.40			17.50		0.742

CR: complete response, PR: partial response, SD: stables disease, PD: progressive disease, CI: confidence interval, ORR: objective response rate.

### sAPE1 was positively associated with APE1 expression in tumor tissue

Although APE1 levels in biopsy tissue showed an inverse correlation with response to platinum-containing chemotherapy, biopsy tissues are not always easy to obtain, which makes it difficult to monitor treatment responsiveness during chemotherapy. Our previous study showed that elevated APE1 protein could be detected in the serum of lung cancer patients, although statistical significance was not reached [14]. In the current study, we sought to measure the chemotherapy-naïve sAPE1 level by using a newly established ELISA protocol. Using a cohort of 52 advanced NSCLC patients randomly picked from the tissue cohort, along with corresponding pre-treatment serum, we explored whether sAPE1 level was associated with its tissue expression. Based on Spearman's correlation analysis, the data show a positive relationship between sAPE1 and tissue expression (r^2^ = 0.639, *p* < 0.001). Intriguingly, the degree of cytoplasmic localization of APE1 is not associated with sAPE1 level based on our analysis.

### Increased APE1 protein level detected in serum of NSCLC patient

**Figure 3:**
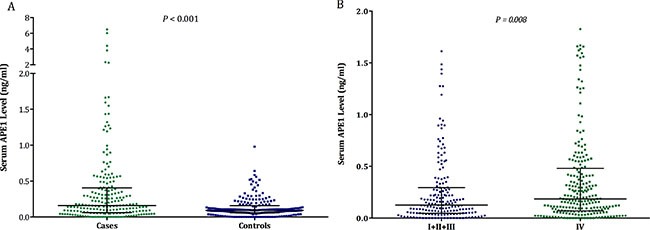
The sAPE1 level in NSCLC patients and healthy subjects assessed by ELISA The sAPE1 level (**A**) between NSCLC cases and controls and (**B**) between NSCLC patients with stage I–III and stage IV.

Following up on the pilot study above, we next tested if sAPE1 was elevated in NSCLC patients by measuring the chemotherapy-naïve sAPE1 level in a cohort of 412 advanced NSCLC patients and 523 healthy donors. Four hundred subjects (1:1) were randomly selected from the whole research set, matched by age and gender (Table 3). Our data revealed a significant elevation of sAPE1 in NSCLC patients compared with controls (Median level: 0.159 vs. 0.091, *p* < 0.001, Figure 3A). The same trend was also observed in stage IV patients compared with other patients with earlier stages (Figure 3B).

**Table 3:** The demographic and clinical characteristics of serum cohort

	Cases (*n* = 200)	Controls (*n*= 200)	*P* value
Age			
Mean ± SD	58.42 ± 9.58	56.98 ± 10.19	1.000
< 60	127 (63.5%)	127 (63.5%)	
> = 60	73 (36.5%)	73 (36.5%)	
Gender			0.765
Male	127 (63.5%)	124 (62.0%)	
Female	73 (36.5%)	76 (38.0%)	
Smoking Status*		0.036	
Non-smoker	107 (53.5%)	86 (43.0%)	
Smoker	93 (46.5%)	114 (57.0%)	
Family History of Cancer			0.663
Negative	171 (85.5%)	174 (87.0%)	
Positive	29 (14.5%)	26 (13.0%)	
Serum APE1 level (ng/ml)			
Median (range)	0.159 (0∼6.48)	0.091 (0∼0.98)	< 0.001

* Two Controls had no exact history of smoking.

**Figure 4:**
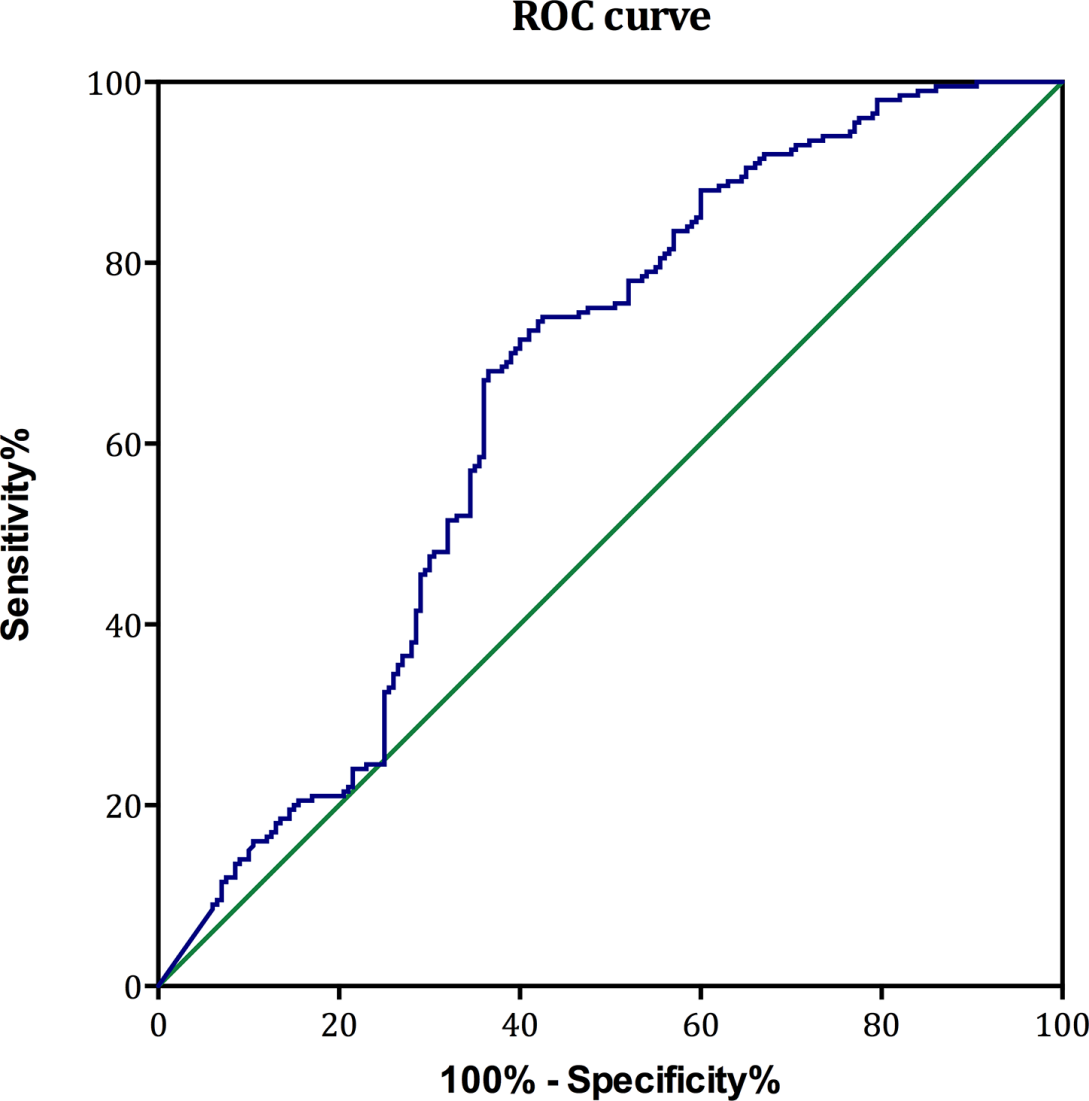
The ROC curves of sAPE1 level for diagnosing NSCLC The diagnostic potentials of quantifying sAPE1 were assessed by ROC curves. The AUC value was 0.653. The cut-off value (0.135 ng/ml) was determined by Youden's index.

The multivariate analysis indicates that chemotherapy-naïve sAPE1 level could be an independent predictive factor for NSCLC (OR: 6.082, 95% CI: 2.278–13.561, *p* < 0.001, Table 4). The area under the curve (AUC) of the receiver operator characteristic (ROC) curve for sAPE1 level was 0.653 (Figure 4). The Youden's index therefore determined the appropriate sAPE1 predictive value to be 0.135 ng/ml. At this level, the sensitivity, specificity and accuracy of sAPE1 for NSCLC diagnosis was 55.58%, 70.75% and 64.06%, respectively. In addition, our analyses displayed an increased diagnostic sensitivity and accuracy of 53.69% and 77.39% for the combination of sAPE1 and CEA levels, compared to 42.61% and 74.81% of CEA alone (Table 4).

**Table 4:** Multivariable logistic regression analysis of APE1 and CEA level in sera of NSCLC

Characteristics	Multivariate Logistic regression analysis*				Detection efficiency		
	Regression coefficient	Odds ratio (OR)	95% Confidential interval (95% CI)	*P* value	Sensitivity	Specificity	Accuracy
sAPE1 level (ng/ml)	1.805	6.082	2.278–13.561	< 0.001	55.58%	70.75%	64.06%
CEA level (ng/ml)	0.252	1.286	1.187–1.394	< 0.001	42.61%	99.81%	74.81%
sAPE1 and CEA combination	0.999	2.716	2.104–3.507	< 0.001	53.69%	95.79%	77.39%

* Adjusted with age, gender, smoking status and family history of cancer.

### Higher sAPE1 level is associated with poor chemotherapeutic outcomes

**Figure 5:**
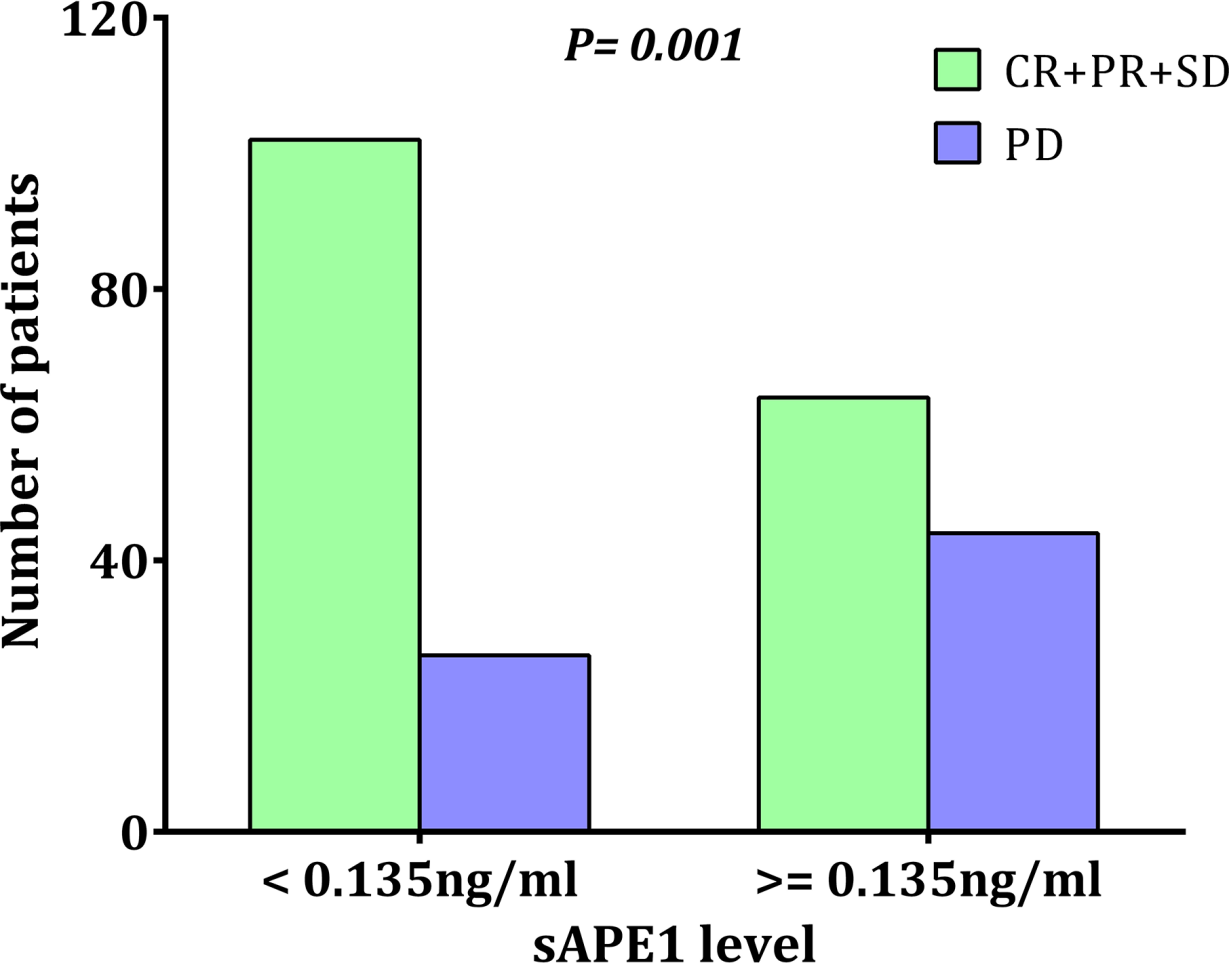
The distribution of treatment responses in different initial sAPE1 level The initial sAPE1 levels were determined before exposure to any chemotherapeutic drugs by ELISA. Comparison of patients with initially low levels (< 0.135 ng/ml) of sAPE1 versus those with high levels (> = 0.135 ng/ml) showed a difference in response to platinum-containing chemotherapy (*p* = 0.001).

Our previous laboratory results indicated that APE1 is a responsive gene for platinum treatment and that deficiency of APE1 in cancer cells increases sensitivity to platinum-based chemotherapy [15]. A chemotherapy-naïve sAPE1 level higher than 0.135 ng/ml was categorized as an elevated level (see above). A higher disease control rate (DCR) was observed in patients with chemotherapy-naïve sAPE1 levels below the cut-off value (79.69% vs. 59.26%, *p* = 0.001, Figure 5). Conversely, elevated sAPE1 is an independent factor for poor NSCLC treatment response (Table 5).

**Table 5:** The association between sAPE1 levels and chemotherapeutic responses and disease outcomes

sAPE1 levels (ng/ml)		N	DCR OR (95% CI)	*P* value	Median	PFS HR (95% CI)	*P* value	Median	OR HR (95% CI)	*P* value
Initial level	< 0.135	128			10.00			29.00		
	> = 0.135	108	2.600 (1.413–4.784)	0.002	6.00	1.421(1.079–1.872)	0.012	29.00		0.210
Change trends	High (H)	58			4.00			18.00		
	Elevated (E)	66	0.332 (0.146–0.753)	0.008	8.00	0.652 (0.445–0.955)	0.028	29.00	0.649 (0.395–1.066)	0.088
	Reduced (R)	50	0.429 (0.183–1.009)	0.052	8.00	0.612 (0.406–0.922)	0.019	38.00	0.510 (0.288–0.902)	0.021
	Low (L)	62	0.188 (0.075–0.471)	< 0.001	12.00	0.449 (0.301–0.670)	< 0.001	32.00	0.497 (0.291–0.849)	0.010

DCR: Disease control rate; PFS: Progression-free survival; OS: Overall survival.

**Figure 6:**
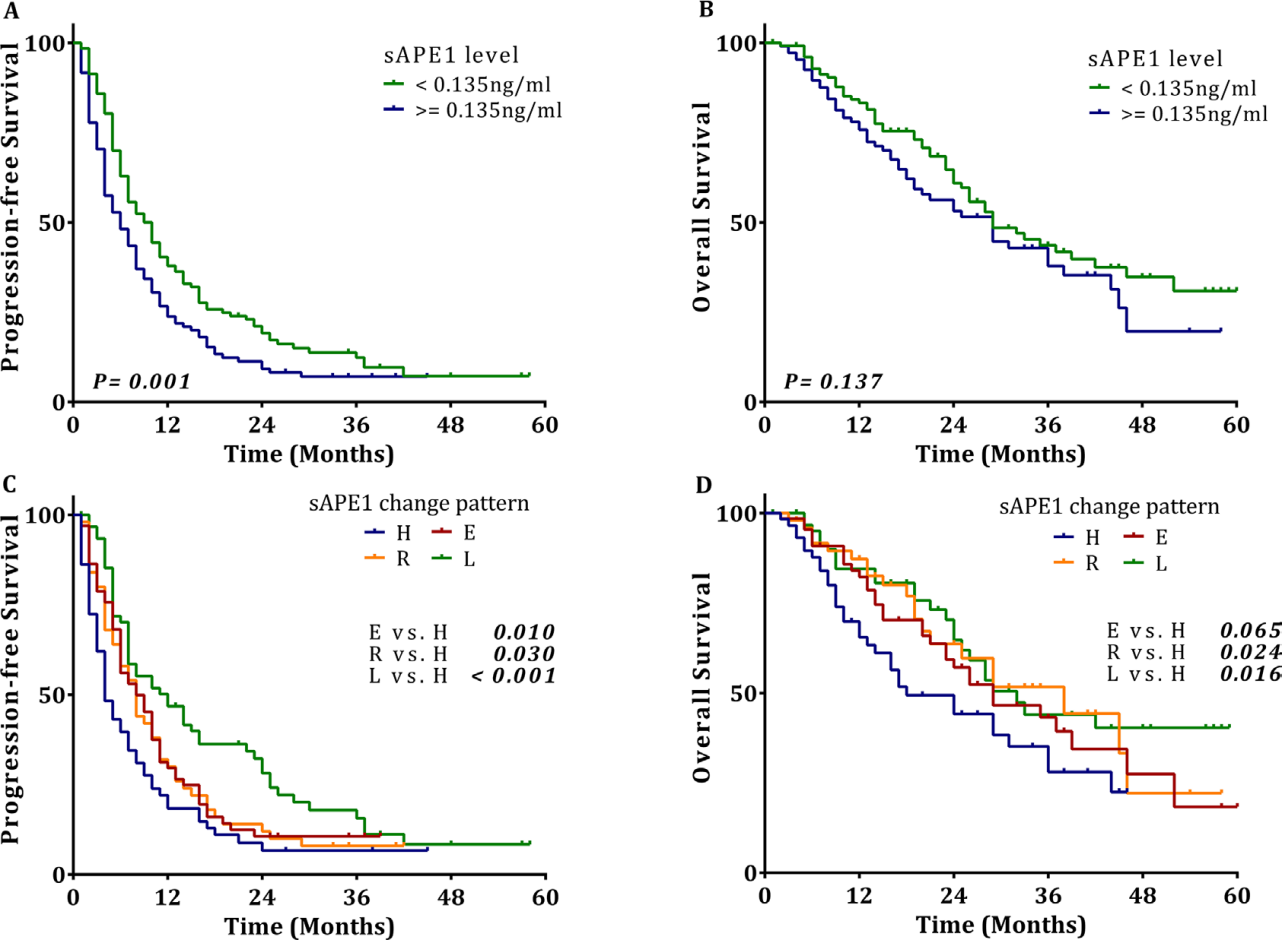
The associations of sAPE1 level and sAPE1 level change pattern with outcomes of NSCLC Kaplan-Meier univariate analysis of (**A**) progression-free survival (10.0 vs. 6.0 months, *p* = 0.001) and (**B**) overall survival (29.0 vs. 29.0 months, *p* = 0.137) for NSCLC patients with a low sAPE1 level compared to those with a high sAPE1 level. (**C**) progression-free survival (E vs. H: 8.0 vs. 4.0 months, *p* = 0.010; R vs. H: 8.0 vs. 4.0 months, *p* = 0.030, L vs. H: 12.0 vs. 4.0 months, *p* < 0.001) and (**D**) overall survival (E vs. H: 29.0 vs. 18.0 months, *p* = 0.065, R vs. H: 38.0 vs. 18.0 months, *p* = 0.024, L vs. H: 32.0 vs. 18.0 months, *p* = 0.016) for NSCLC patients with sAPE1 change patterns E, R, L compared to those with sAPE1 change pattern H.

Survival analysis revealed that patients with chemotherapy-naïve sAPE1 levels below the cut-off value exhibit an increased PFS compared to those with an elevated level (6.0 vs. 10.0 months, *p* = 0.001, Figure 6A), although low serum levels were not beneficial for OS (29.0 vs. 29.0 months, Figure 6B). Again, elevated sAPE1 level is an independent risk factor for PFS in NSCLC receiving platinum-containing chemotherapy (HR: 1.421, 95% CI: 1.079–1.872, *p* = 0.012, Table 5).

### The pattern of sAPE1 level alteration during chemotherapy is associated with the therapeutic response of NSCLC

To explore the possibility of monitoring therapeutic efficacy using sAPE1 changes during chemotherapy, we tracked the sAPE1 level of 236 cases of NSCLC patients at ∼1 month after the sixth cycle. All subjects were categorized into these four groups: group L are those patients whose sAPE1 level started and remained below the cut off (< 0.135 ng/ml) during the treatment; group E had an initially low sAPE1 level, which was elevated over the cut-off after treatment; group H consists of patients whose sAPE1 level started and remained above the cut off (> = 0.135 ng/ml) during the treatment; and group R includes those patients that had an initially high sAPE1 level, which was reduced to below the cut-off. Compared with group H, the other groups demonstrated significantly decreased risks of progression in disease after treatment (HR: 0.449, 95% CI: 0.301–0.670, *p* < 0.001 for group L; HR: 0.612, 95% CI: 0.406–0.922, *p* = 0.019 for group R; HR: 0.652, 95% CI: 0.445–0.955, *p* = 0.028 for group E). In the survival analysis, compared to group H, all the other groups exhibited a distinct increase in median PFS (Figure 6C). The three groups also had a longer median OS compared with group H (Figure 6D). Moreover, the multivariate Cox regression confirmed that compared with group H, the other groups were independent protective factors for PFS; and while only groups L and R were associated with better outcomes for OS, no statistical difference was observed between groups E and H (Table 5). These results strongly suggest that monitoring sAPE1 level could be a potential strategy to predict efficacy of platinum-containing chemotherapy in NSCLC patients.

## DISCUSSION

Unlike the targeted drugs utilized in the treatment of NSCLC, such as the EGFR-TKIs and ALK inhibitors, chemotherapeutic agents, including platinum, are usually applied to cancer patients without any specific gene target in mind, but instead are based on established response rates in previous trials. While collective efforts have been made to identify predictive biomarkers for the efficacy of chemotherapy, little progress have been made in this arena, particularly as it relates to DNA repair [3]. In the current study, we observed that in the tumor tissue, APE1 protein, an essential DNA repair enzyme in the BER pathway, could be a promising predictive biomarker for the responses of NSCLC patients receiving platinum-containing chemotherapy. Moreover, this previously reported nuclear protein was detected in the serum of NSCLC patients, and its level in the serum was directly correlated with the level measured in cancer tissue [12, 16, 17]. In addition, we describe herein, for the first time to our knowledge, that alteration of sAPE1 after chemotherapy is significantly associated with response and PFS of platinum-containing chemotherapy. Thus, our data indicate that monitoring the sAPE1 level could be an auxiliary strategy for physicians to predict the response to chemotherapy.

The cytotoxicity of platinum drugs is associated with their ability to crosslink DNA strands, which can lead to defective DNA replication and increased strand breaks [18]. Therefore, it is reasonable to explore biomarkers associated with platinum resistance within DNA repair pathways [19], such as the excision repair cross-complementation group 1 (ERCC1), which is the most studied candidate for predicting platinum-containing chemotherapy response. ERCC1 protein plays an essential role in nucleotide excision repair (NER), in which it cleaves DNA structures near the site of the platinum–DNA adduct, thereby allowing eventual removal of the lesion. Based on the hypothesis that DNA repair mechanisms play a critical role in dictating overall genotoxic agent responsiveness, retrospective and prospective studies have been performed to explore the clinical correlation between ERCC1 expression (at both the RNA and protein level, as well as with respect to SNPs) with prognosis and relevant chemotherapy response. Previous clinical trials, however, have found limited, if not negative, predictive effects of ERCC1 expression (as measured by IHC in tumor tissue) on the efficacy of platinum-containing chemotherapy [20]. Our current study also analyzed the correlation between ERCC1 expression in biopsy tissue and efficacy of platinum-containing chemotherapy in the same cohort in which APE1 expression was measured. In agreement with the majority of previous studies, we found that although ERCC1 expression is inversely associated with ORR for the platinum-containing chemotherapy receivers, the responders did not have a longer PFS, which is considered to be a key efficacy marker. In addition, ERCC1 expression is inversely correlated with OS, making it a poor predictive marker overall (Table 6).

**Table 6:** Association of ERCC1 tissue expression with the outcomes of NSCLC receiving chemotherapy

		Treatment response					Progression-free survival			Overall survival	
	Number of cases	CR + PR	SD + PD	Multivariable Logistic analysis		Months	Multivariable COX regression		Months	Multivariable COX regression	
				ORR (95% CI)	*P* value		HR (95% CI)	*P* value		HR (95% CI)	*P* value
ERCC1 tissue expression											
Low	94	39	55	41.49		9.8			14.4		
High	78	16	62	20.51	3.094 (1.351–7.086)	6.8	2.917 (2.160–3.939)	< 0.001	10.3	2.460 (1.613–3.753)	< 0.001

CR: complete response, PR: partial response, SD: stables disease, PD: progressive disease, CI: confidence interval, ORR: objective response rate.

Our analysis conducted here found that high APE1 tissue expression is significantly correlated with reduced ORR, and in turn a shorter PFS, suggesting APE1 level could be an effective predictive biomarker for chemotherapy. However, APE1 is not associated with OS according to our analyses, implying more complex roles of APE1 in the evolution of NSCLC, perhaps reflecting the diversity of its biological functions. As OS could be affected by many factors, including side effects of chemotherapy, we postulate that APE1 plays a role in coping with the effects of reactive oxygen species produced by platinum agents, as described previously [21]. Future studies are needed to analyze the role of APE1 in affecting subsequent treatment responses after standard chemotherapy.

In the present study, we have described a role for sAPE1 in evaluating the effectiveness of platinum-containing chemotherapy for NSCLC patients. The APE1 protein was initially reported to be present in serum by our group, and its level, although not statistically significant, was observed to be elevated in NSCLC patients [14]. In our current study, we report that APE1 serum level was significantly elevated in NSCLC patients, in agreement with reports for other cancers including bladder cancer [17]. The discrepancy with our prior work could be, we postulate, due to the following circumstances: a) the overall APE1 serum level was low and the sensitivity of the previous ELISA assay was too low to distinguish a difference between the levels in cancer patients from even lower levels in healthy donors; b) as APE1 level is altered by chemotherapeutic agents, only pretreatment or chemotherapy-naïve serum samples were analyzed in the current study, which should represent the cancer-associated level; or c) simply owing to the larger sample size in the present study.

Our results show that APE1 is a strong biomarker for NSCLC, and that combining it with a previously recognized biomarker for NSCLC, CEA, further enhances the power of both, but the use of sAPE1 as a biomarker for other cancers, such as small cell lung cancer (SCLC), needs to be explored. We found that the pretreatment level of sAPE1 has a similar ability to predict chemotherapy efficacy as compared to its tissue level. More importantly, monitoring sAPE1 alteration after chemotherapy is also beneficial for prediction of the response, making it a more feasible and informative biomarker than ERCC1 tissue expression. Intriguingly, our data unexpectedly revealed that patients with low initial sAPE1 level (below cut-offvalue), which increased to above the cut-off value during chemotherapy, had the same PFS as patients with a high initial and then reduced level during chemotherapy, suggesting that the initial level as well as changes in sAPE1 during the process of chemotherapy are important to therapeutic efficacy. Moreover, our study highlights that low post-treatment sAPE1 levels are associated with longer OS, irrespective of the initial level of sAPE1; further emphasizing that monitoring of sAPE1 could predict the efficacy of chemotherapy.

As a protein mainly localized in the nucleus, the source of sAPE1, whether it be from normal or cancer cells, is currently unknown and the focus of ongoing investigations. In our present study, we show a direct correlation between serum and tissue APE1 levels in the cohort we analyzed. It is unlikely that sAPE1 originates from passive release by a necrotic process, because we analyzed the samples from untreated subjects, and laboratory experiments showed that supernatant APE1 levels were nearly unchanged when cultured A549 cells were treated with increased doses of cisplatin (data not shown). This observation makes it tenable that the presence of APE1 in serum is due to an active process, such as secretion or through an exosomal pathway. Our laboratory data indicate that when detecting APE1 in serum treated with 1% Triton-X100 detergent, APE1 levels remain unchanged, suggesting that APE1 exists in serum likely as the protein itself, and not within vesicles, such as exosomes. However, APE1 is not a typical secretory protein as it lacks classic secretory signals based on protein sequence analysis. Intriguingly, there are reports showing that APE1 is secreted under specific stimuli [22, 23]. The secretion of APE1 was associated with its cytoplasmic translocation according to these reports; however, we failed to build a positive connection between sAPE1 and its cytoplasmic distribution. Future work is required to delineate the secretory mechanisms of APE1 and its functional purpose in the circulation, if any.

## MATERIALS AND METHODS

### Study design and patient population

A total of 172 stage IIIB and IV NSCLC patients were enrolled for APE1 tissue assessment. All tumors were pathologically confirmed during August 2007 to August 2012 in Daping Hospital of the Third Military Medical University (Chongqing, China). The following criteria were required for inclusion in the study: 1) patients must have received at least two cycles of first line platinum-based chemotherapy with a measurable tumor response, and 2) the paraffin-embedded tissue samples must be available. All patients received the platinum-based doublets chemotherapy, i.e., TP regimen (Platinum & Paclitaxel) or GP regimen (Platinum & Gemcitabine). The entire treatment protocol was devised to follow the NCCN guidelines. Tumor response was evaluated after every two cycles of chemotherapy and was classified using RECIST (version 1.1). Demographic and clinical data were obtained by office interviews or telephone visit. The histological type, tumor stage, and TNM classification were analyzed according to the World Health Organization. The whole cohort was followed up every 6 weeks until the disease progressed and then every 3 months until death. The median follow-up time was 11 months (range, 1–43 months) measured from the onset of chemotherapy.

**Figure 7:**
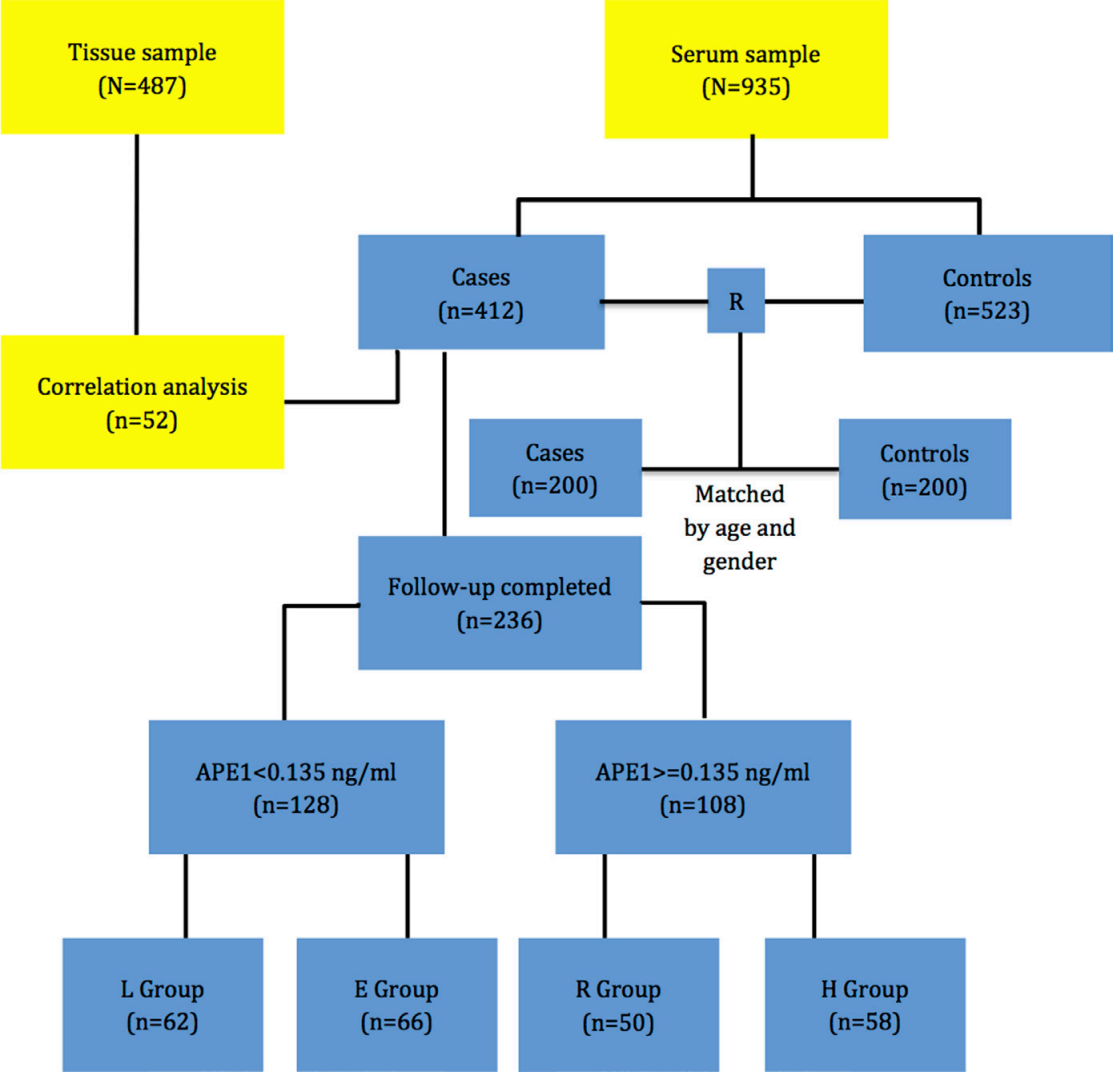
The research protocol 172 pathologically confirmed NSCLC patients with full follow-up information were enrolled, and their APE1 expression in tissue was evaluated by IHC. For serum research, 412 confirmed NSCLC patients and 523 cancer-free controls were included. 400 subjects (1:1 for Cases: Controls) were randomly picked from the whole set and matched by age and gender for diagnosis analysis. Patients completing at least 6 months therapy with full follow-up information were subsequently enrolled into prognostic analysis. Patients were divided into two groups: those with initial serum APE1 level lower than cut-off value (< 0.135 ng/ml) and those with higher than cut-off value (> = 0.135 ng/ml). The pattern of sAPE1 alteration was observed in the first 6 months and categorized into 4 groups: group L represents patients whose sAPE1 level both began and remained below the cut-off during the first 6 months of treatment; group E represents patients with initially low sAPE1 levels, which became elevated above the cut-off after treatment; group H represents patients whose sAPE1 level both began and remained above the cut-off (> = 0.135 ng/ml) during the first 6 months of treatment; and group R represents patients with an initially high sAPE1 level, which was reduced to below the cut-off.

For serum research, we enrolled 412 confirmed NSCLC patients and 523 cancer-free controls randomly picked from those determined to be healthy by examination. All samples were obtained within a five-year period from January 2010 to December 2014. The demographic and clinical data were obtained and described previously. Total 400 subjects (200 cases: 200 controls) were picked out randomly for diagnosing analysis from the whole research cohort matched by age and gender. Patients who completed at least 6 cycles of standard treatments and follow-ups were subsequently enrolled in the analysis. The change in sAPE1 was observed in the first 6 months from the onset of treatments. To further investigate the association between sAPE1 level and disease outcomes, four patterns of alteration were defined and their differences in DCR, PFS and OS were analyzed (Figure 7). The whole protocol was approved by the Ethics Committee of Daping Hospital, Third Military Medical University, China and written informed consents were obtained from all patients and healthy controls.

### Immunohistochemical evaluation of paraffin-embedded tumor tissues

The IHC was performed to determine APE1 expression in NSCLC tissues described previously [24]. Protein was scored for four categories, i.e., score 0, no expression in tumor cells; score 1+, faint/barely perceptible partial nuclear expression in < 10% of tumor cells; score 2+, weak to moderate expression of the entire nucleus in > 10% of tumor cells; and score 3+, strong expression of the entire nucleus in > 10% of tumor cells. Score 2+/3+ was defined as high expression, while score 0/1+ as low expression. We have described this procedure in detail in Supplementary Methods.

### Purification of recombinant APE1 protein

Untagged, human recombinant APE1 protein was overexpressed from pETApe, according to the protocol described previously [25]. The supernatant after centrifugation was then passed through the Q FF column containing the balance buffer (50 mM Hepes-KOH pH 7.5, 5% glycerol, 50 mM KCl and 1 mM DTT) and wash buffer (2 M NaCl) to remove impurities. Then it passed through the SP FF column containing the same balance buffer but a different wash buffer (50mM Hepes-KOH pH 7.5, 5% glycerol, 700 mM KCl and 1 mM DTT) with sequentially increasing concentrations of 10%, 20%, 30%, 50%, 80%, and 100% 2 M NaCl. After completing the protocol, purified APE1 protein was obtained. A sample of the protein was denatured and detected by electrophoresis and the final concentration was determined by Lowry assay to be 3.1 mg/ml.

### Serum sample extraction and sAPE1 level detection

Blood samples were collected before any treatment and at ∼1 month after 6 cycles of chemotherapy for the NSCLC patients or at the time of routine examination for healthy subjects. The serum samples were isolated routinely and subjected to ELISA immediately or stored at −80°C for further analysis. Detailed protocol for ELISA can be obtained through Supplementary Methods.

### Statistics

The quantitative data was analyzed by non-parametric test, while the categorical data was analyzed by chi-square analysis or Fisher's exact test. The Spearman's correlation test was performed for relationship analysis. The logistic regression model was constructed to evaluate the relationship of sAPE1 level to NSCLC diagnosis. The receiver operating characteristic (ROC) curve was drawn to estimate the diagnostic value of sAPE1. Kaplan-Meier analysis and Cox proportional hazard regression model were performed to evaluate the association between sAPE1 level and NSCLC treatment outcomes.

## SUPPLEMENTARY MATERIALS FIGURE AND TABLE



## References

[1] Kandoth C, McLellan MD, Vandin F, Ye K, Niu B, Lu C, Xie M, Zhang Q, McMichael JF, Wyczalkowski MA, Leiserson MD, Miller CA, Welch JS (2013). Mutational landscape and significance across 12 major cancer types. Nature.

[2] Khanna A (2015). DNA Damage in Cancer Therapeutics: A Boon or a Curse?. Cancer Res.

[3] Passaro A, Palazzo A, Trenta P, Mancini ML, Morano F, Cortesi E (2012). Molecular and clinical analysis of predictive biomarkers in non-small-cell lung cancer. Current Medicinal Chemistry.

[4] Jalal S, Earley JN, Turchi JJ (2011). DNA repair: from genome maintenance to biomarker and therapeutic target. Clin Cancer Res.

[5] Winter SF, Minna JD, Johnson BE, Takahashi T, Gazdar AF, Carbone DP (1992). Development of antibodies against p53 in lung cancer patients appears to be dependent on the type of p53 mutation. Cancer Res.

[6] Laurent-Puig P, Lubin R, Semhoun-Ducloux S, Pelletier G, Fourre C, Ducreux M, Briantais MJ, Buffet C, Soussi T (1995). Antibodies against p53 protein in serum of patients with benign or malignant pancreatic and biliary diseases. Gut.

[7] Tomkiel JE, Alansari H, Tang N, Virgin JB, Yang X, VandeVord P, Karvonen RL, Granda JL, Kraut MJ, Ensley JF, Fernandez-Madrid F (2002). Autoimmunity to the M(r) 32,000 subunit of replication protein A in breast cancer. Clin Cancer Res.

[8] Zhu Q, Han SX, Zhou CY, Cai MJ, Dai LP, Zhang JY (2015). Autoimmune response to PARP and BRCA1/BRCA2 in cancer. Oncotarget.

[9] Okada T, Noji S, Goto Y, Iwata T, Fujita T, Okada T, Matsuzaki Y, Kuwana M, Hirakata M, Horii A, Matsuno S, Sunamura M, Kawakami Y (2005). Immune responses to DNA mismatch repair enzymes hMSH2 and hPMS1 in patients with pancreatic cancer, dermatomyositis and polymyositis. Int J Cancer.

[10] Fernandez Madrid F (2005). Autoantibodies in breast cancer sera: candidate biomarkers and reporters of tumorigenesis. Cancer Lett.

[11] Balogh GA, Mailo DA, Corte MM, Roncoroni P, Nardi H, Vincent E, Martinez D, Cafasso ME, Frizza A, Ponce G, Vincent E, Barutta E, Lizarraga P (2006). Mutant p53 protein in serum could be used as a molecular marker in human breast cancer. Int J Oncol.

[12] Li M, Wilson DM (2014). Human apurinic/apyrimidinic endonuclease 1. Antioxid Redox Signal.

[13] Katsumata Y, Kawaguchi Y, Baba S, Hattori S, Tahara K, Ito K, Iwasaki T, Yamaguchi N, Oyama M, Kozuka-Hata H, Hattori H, Nagata K, Yamanaka H, Hara M (2011). Identification of three new autoantibodies associated with systemic lupus erythematosus using two proteomic approaches. Mol Cell Proteomics.

[14] Dai N, Cao XJ, Li MX, Qing Y, Liao L, Lu XF, Zhang SH, Li Z, Yang YX, Wang D (2013). Serum APE1 autoantibodies: a novel potential tumor marker and predictor of chemotherapeutic efficacy in non-small cell lung cancer. PLoS One.

[15] Wang D, Xiang DB, Yang XQ, Chen LS, Li MX, Zhong ZY, Zhang YS (2009). APE1 overexpression is associated with cisplatin resistance in non-small cell lung cancer and targeted inhibition of APE1 enhances the activity of cisplatin in A549 cells. Lung cancer.

[16] Jin SA, Seo HJ, Kim SK, Lee YR, Choi S, Ahn KT, Kim JH, Park JH, Lee JH, Choi SW, Seong IW, Jeon BH, Jeong JO (2015). Elevation of the Serum Apurinic/Apyrimidinic Endonuclease 1/Redox Factor-1 in Coronary Artery Disease. Korean Circ J.

[17] Shin JH, Choi S, Lee YR, Park MS, Na YG, Irani K, Lee SD, Park JB, Kim JM, Lim JS, Jeon BH (2015). APE1/Ref-1 as a Serological Biomarker for the Detection of Bladder Cancer. Cancer Res Treat.

[18] Bonanno L, Favaretto A, Rosell R (2014). Platinum drugs and DNA repair mechanisms in lung cancer. Anticancer Res.

[19] Postel-Vinay S, Vanhecke E, Olaussen KA, Lord CJ, Ashworth A, Soria JC (2012). The potential of exploiting DNA-repair defects for optimizing lung cancer treatment. Nat Rev Clin Oncol.

[20] Friboulet L, Olaussen KA, Pignon JP, Shepherd FA, Tsao MS, Graziano S, Kratzke R, Douillard JY, Seymour L, Pirker R, Filipits M, Andre F, Solary E (2013). ERCC1 isoform expression and DNA repair in non-small-cell lung cancer. N Engl J Med.

[21] Kelley MR, Jiang Y, Guo C, Reed A, Meng H, Vasko MR (2014). Role of the DNA base excision repair protein, APE1 in cisplatin, oxaliplatin, or carboplatin induced sensory neuropathy. PLoS One.

[22] Choi S, Lee YR, Park MS, Joo HK, Cho EJ, Kim HS, Kim CS, Park JB, Irani K, Jeon BH (2013). Histone deacetylases inhibitor trichostatin A modulates the extracellular release of APE1/Ref-1. Biochem Biophys Res Commun.

[23] Lee YR, Kim KM, Jeon BH, Choi S (2015). Extracellularly secreted APE1/Ref-1 triggers apoptosis in triple-negative breast cancer cells via RAGE binding, which is mediated through acetylation. Oncotarget.

[24] Li Z, Qing Y, Guan W, Li M, Peng Y, Zhang S, Xiong Y, Wang D (2014). Predictive value of APE1, BRCA1, ERCC1 and TUBB3 expression in patients with advanced non-small cell lung cancer (NSCLC) receiving first-line platinum–paclitaxel chemotherapy. Cancer Chemother Pharmacol.

[25] Erzberger J (1998). Elements in abasic site recognition by the major human and Escherichia coli apurinic/apyrimidinic endonucleases. Nucleic Acids Res.

