# Supplementary material for: Serum APE1 as a predictive marker for platinum-based chemotherapy of non-small cell lung cancer patients
Source: Oncotarget. 2016 Nov 2;7(47):77482–94. doi: 10.18632/oncotarget.13030 (PMC5340230; doi:10.18632/oncotarget.13030)
Supplement: Supplementary file 1 [file oncotarget-07-77482-s001.pdf]

Serum APE1 as a predictive marker for platinum-based chemotherapy of non-small cell lung cancer patients

Supplementary Materials

SUPPLEMENTARY MATERIALS (PATIENTS AND METHODS)

Study subjects

Our research protocols were developed based on two main components: APE1 levels in tissue and in serum. The 172 NSCLC patients were pathologically confirmed during August 2007 to August 2012 in Daping Hospital of the Third Military Medical University (Chongqing, China). The following criteria were required for inclusion in the study: 1) patients must have received at least two cycles of first line platinum-based chemotherapy with a measurable tumor response, and 2) the paraffin-embedded tissue samples must be available. The entire treatment protocol was devised to follow the NCCN guidelines (version 7). Tumor response was evaluated after every two cycles of chemotherapy and was classified using RECIST (version 1.1). Demographic and clinical data were obtained by office interviews or telephone visit. The histological type, tumor stage, and TNM classification were analyzed according to the World Health Organization. The whole cohort was followed up every 6 weeks until the disease progressed and then every 3 months until death. The median follow-up time was 11 months (range, 1–43 months) measured from the onset of chemotherapy. All serum samples were obtained within a five-year period from January 2010 to December 2014. The demographic and clinical data were obtained and described previously. Patients who completed at least 6 cycles of standard treatments and follow-ups were subsequently enrolled in the prognostic analysis. The change in sAPE1 was observed in the first 6 months from the onset of treatments. To further investigate the association between sAPE1 level and disease outcomes, four patterns of alteration were defined and their differences in DCR, PFS and OS were analyzed.

Immunohistochemical evaluation of paraffin-embedded tumor tissues

All paraffin tissue blocks were incidental (residual) samples of bronchoscopy or fine needle aspiration biopsies for diagnostic purposes. Sections from paraffin-embedded tumors were incubated overnight with APE1 monoclonal antibody [Novus, Littleton, CO, USA]), then incubated with goat anti-mouse secondary antibody (Pierce, Rockford, IL, USA) for 30 minutes at 37°C. Antigen–antibody complexes were visualized by incubation with 3,3′-diaminobenzidine (DAB) substrate and counterstained with diluted Harris hematoxylin. The immunostained tissue sections were evaluated and scored under a light microscope independently by two pathologists in a blinded fashion.

Purification of recombinant APE1 protein

The supernatant after centrifugation was then passed through the Q FF column containing the balance buffer (50 mM Hepes-KOH pH 7.5, 5% glycerol, 50 mM KCl and 1 mM DTT) and wash buffer (2 M NaCl) to remove impurities. Then it passed through the SP FF column containing the same balance buffer but a different wash buffer (50 mM Hepes-KOH pH 7.5, 5% glycerol, 700 mM KCl and 1 mM DTT) with sequentially increasing concentrations of 10%, 20%, 30%, 50%, 80%, and 100% 2 M NaCl. After completing the protocol, purified APE1 protein was obtained.

sAPE1 level detection

A newly established ELISA kit was used to detect sAPE1 level and carried out as follows: 1) 100 µl/well of standards and serum samples were added into the prepared 96-well plates and incubated at 37ºC for 1 hour; 2) the plate was washed 5 times using 200 µl/well of washing buffer, then incubated with 100 µl/well of horseradish peroxidase conjugate at 37ºC for 1 hour; 3) After washing the plate 5 times, 100 µl/well of TMB substrate solution was incubated for 15 min at 37ºC; 4) The enzymatic reaction was stopped by a prepared stop solution (50 µl/well) and then optical density (OD) was measured by microplate spectrophotometry at the reference wavelength (450 nm). The analysis sensitivity of the ELISA kit was 0.02 ng/ml. Satisfactory within-batch and between-batches precisions were achieved with coefficient of variation (CV) less than 10% and 15%, and the average recoveries obtained were in the range of 90.6–108.3%.

Supplementary Table S1: The demographic and clinical characteristics of whole serum cohort

	Cases (*n* = 412)	Controls (*n* = 523)	*P* value
Age			
Mean ± SD	59.67 ± 10.83	44.87 ± 13.43	< 0.001
< 60	214 (51.90%)	437 (83.60%)	
> = 60	198 (48.10%)	86 (16.40%)	
Gender			< 0.001
Male	291 (70.60%)	298 (57.00%)	
Female	121 (29.40%)	225 (43.00%)	
Smoking Status*			< 0.001
Non-smoker	196 (47.60%)	335 (64.10%)	
Smoker	216 (52.40%)	186 (35.60%)	
Family History of Cancer			0.558
Negative	355 (86.20%)	456 (87.20%)	
Positive	57 (13.80%)	65 (12.40%)	
Serum APE1 level (ng/ml)			
Median (range)	0.16 (0~6.48)	0.09 (0~2.04)	< 0.001
Pathology			
Adenocarcinoma	226 (54.90%)	/	
Squamous cell carcinoma	125 (30.30%)	/	
Other	61 (14.80%)	/	
Stage			
I+II+III	176 (42.70%)	/	
IV	232 (56.30%)	/	
Unknown	4 (1.00%)	/	

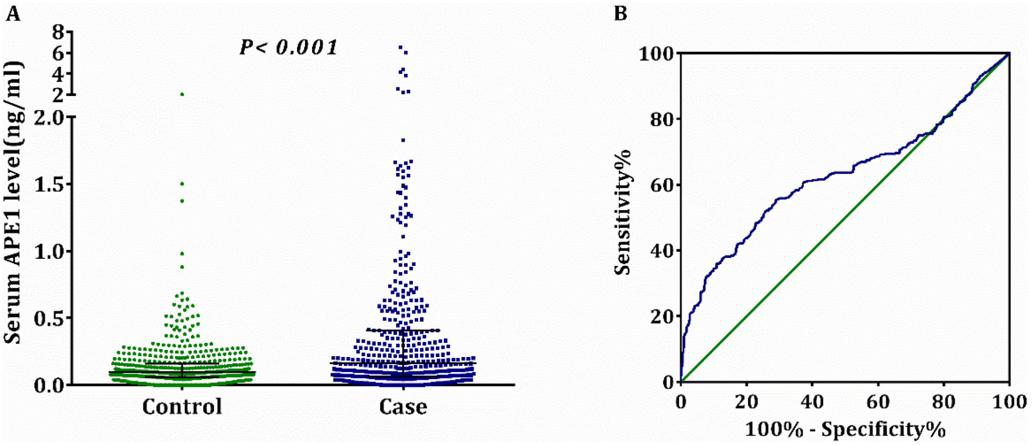

Supplementary Figure S1: The serum APE1 level in NSCLC patients and healthy subjects and the ROC of serum APE1 level for diagnosing NSCLC. (**A**) The distributions of sAPE1 levels in two groups; (**B**) The ROC curve of sAPE1 in diagnosing NSCLC.
